# Reconstructing the spatial and temporal dynamics of Ecuador’s artisanal small-scale fisheries from fishers’ perspective

**DOI:** 10.1371/journal.pone.0338495

**Published:** 2025-12-23

**Authors:** César R. Peñaherrera-Palma, Nicole S. Chinacalle-Martínez, Wendy Gómez Zamora, S. Pamela Jaramillo-Vallejo, Jean P. López-Macías, Omar Safady-Mendoza, M. Estefanía Bravo-Ormaza

**Affiliations:** 1 MigraMar, Bodega Bay, California, United States of America; 2 Carrera de Biología, Facultad de Ciencias de la Vida y Tecnologías, Universidad Eloy Alfaro de Manabí, Av. Circunvalación, Vía a San Mateo, Manta – Manabí, Ecuador; 3 Departamento de Ciencias del Mar y Biología Aplicada, Universidad de Alicante, San Vicente del Raspeig s/n, Alicante, España; 4 Centro Interdisciplinario de Ciencias Marinas, Av. Instituto Politécnico Nacional s/n, Col. Playa Palo de Santa Rita, La Paz, Baja California Sur, México; University of Mississippi, BRAZIL

## Abstract

Despite their crucial role in livelihoods and commercial activities in coastal communities, numerous artisanal fisheries are often underreported and underestimated because of the absence of proper monitoring programs since they started. This lack of data hinders the assessment of the spatial dynamics and status of the resources these fisheries have targeted over time. This is especially concerning, considering the severe overexploitation faced by many coastal resources. Using tools to assess local ecological knowledge, the present study summarizes the perceptions of artisanal fishers regarding spatial and temporal changes in their fishing grounds across nearshore and offshore coastal environments in Ecuador, South America. From April to September 2023, 1,366 fishers from 22 fishing villages were interviewed about the state of their resources and how their fishing grounds and catches changed over the decades of the 1980s, 1990s, 2000s, and 2010s. We employed a semi-quantitative virtual abundance change framework to convert categorical trend values into numerical data and to associate these with the fishing grounds described by each fisher per decade. Almost all fishers interviewed (approximately 99%) indicated that fishing yields have declined significantly, that the number of targeted species has increased, and that their fishing areas have expanded and shifted farther from the coast. Specifically, the overall perceived trends indicate that catches in the 2010s were 30% lower than in the pre-1980s and approximately 59.5% lower than in the 2000s. While there was an important increase in the distance to the port of origin through the decades, the core fishing grounds (where 60–100% of fishers operate) were located near the coast within the first 8 nautical miles. The results of this assessment highlight the importance of collaboration between fishers and scientists in reconstructing the historical dynamics of under-assessed coastal fisheries.

## Introduction

Artisanal fishing is an ancestral activity primarily conducted in coastal areas, serving as the main source of livelihood and commercial activity for artisanal fishers [1]. The revenue generated from artisanal fishing not only fulfills the food needs of fishers but also accounts for approximately half of the global catch volume. This is particularly significant considering that 90% of fishers worldwide are engaged in artisanal fishing [2]. In recent years, the importance of artisanal fishing has gained recognition due to persistently low levels of human development and high rates of relative poverty in these communities [3]. This contrasts with industrial fishing, a sector whose continuous expansion has created unequal competition for resources and market access due to substantial technological and operational advantages over artisanal fishing [4]. This disparity has also influenced management and regulatory efforts, as the industrial sector (equipped with more advanced technology) produces more comprehensive data [5]. Consequently, fisheries management has predominantly relied on information provided by the industrial sector [6], further marginalizing the evaluation and decision-making processes based on artisanal fishing data [7].

The strategic location of Ecuador near the Humboldt upwelling system (originating in Peru) and above the Cromwell upwelling system (originating west of the Galapagos Islands) has allowed the country to develop a significant fishing industry. It is estimated that the artisanal fleet alone generates a national market value of nearly USD 200 million and an international market value of nearly USD 364 million [8]. Although dominated by pelagic drifting longlines, artisanal fisheries employ various types of fishing gear, such as gill nets (e.g., surface, bottom, and trammel nets), hooks (e.g., bottom-set longlines, and hand lines), spearfishing, and hand collection [9]. The predominant vessels are wooden and fiberglass boats [10], which are used to harvest whitefish, mollusks, and crustaceans [11]. Based on the targeted resources and harvesting methods, artisanal fishing is divided into three categories: manual harvesting, coastal fishing, and oceanic fishing [12], where coastal waters are defined as those extending from the coastline up to 70–80 nautical miles and encompassing waters less than 200 meters deep [13]. Since 1990, Ecuador has designated an exclusive zone for artisanal fishers within the first eight nautical miles from the coast [14]. Additionally, there is a reserved zone to protect hydrobiological resources and support specific artisanal extractive activities, which extends from the continental shoreline to the first nautical mile [15].

The management of the artisanal fishing sector in coastal Ecuador faces significant challenges due to inadequate monitoring and data gaps. While fishery inspectors have recorded catches since the 1980s [16], monitoring is largely restricted to major ports and longline vessels targeting pelagic species [8], leaving inshore coastal fisheries poorly documented. The lack of comprehensive spatial and temporal data on artisanal fishing activities, particularly for the ~ 40,000 unequipped vessels [17], hinders effective fisheries management and assessment of long-term dynamics [18,19]. The limited adoption of surveillance technologies like Vessel Monitoring Systems (VMS) and Automatic Identification Systems (AIS) on artisanal vessels, due to high costs and installation challenges on small boats [20], results in only 40% of activities being monitored [18]. Additionally, existing data are often inaccessible or lack the resolution needed for long-term detailed analysis [19].

Leveraging local ecological knowledge (LEK) from artisanal fishers offers a practical solution to bridge these data gaps. LEK refers to the understanding and insights about ecological systems that people develop through direct, long-term interaction with their local environment [21]. Transmitted and shared within communities, this knowledge encompasses beliefs and practices related to how ecosystems function and are used by local populations who are in constant interaction with nature. LEK has been applied in challenging scenarios to evaluate the perception of resource dynamics and to obtain quantitative data. For instance, it has been used to assess connectivity among multiple habitats in tropical seascapes and to evaluate the consistency between LEK and conventional scientific knowledge [22]; to support biodiversity conservation by estimating historical shark abundance within marine protected areas [23]; and to document the spatial and temporal patterns of recreational and subsistence fishing through surveys and hand-drawn fishing maps [24]. As such, LEK can provide insights into spatial dynamics, fishing practices, and ecological changes, thus becoming a critical tool to enable a more comprehensive understanding of inshore fisheries and to support the sustainable management of data-poor scenarios [25–27].

In recent years, it is believed that artisanal fishing along the Ecuadorian coast has undergone shifts in its spatial and temporal dynamics. This hypothesis is supported by localized reports of resource depletion within traditional fishing grounds [10], increased insecurity due to criminal organizations [28,29], increasing economic and social pressures pushing fishers toward illegal activities [30], poor technological and maritime surveillance, changes in the number of active fishers, and insufficient investment and regulatory control [31]. Here, we applied a LEK-based research approach using surveys conducted in fishing villages to i) analyze unmonitored catch trends; ii) examine the spatiotemporal dynamics of fishing grounds; and iii) identify, based on fishers’ perceptions, the factors likely influencing catch levels and the dynamics of fishing grounds in Ecuador’s coastal artisanal fisheries since the 1980s.

## Materials and methods

### Study area

**Fig 1:**
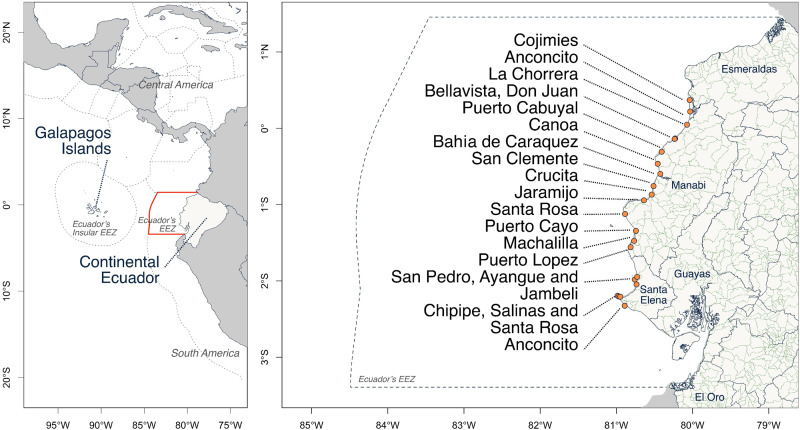
Location of the study area in relation to South America and coastal Ecuador. Administrative divisions and exclusive economic zones (EEZ) are indicated by dotted lines; province boundaries by blue lines; and canton boundaries by green lines. Basemaps: Political and administrative division of Ecuador [32], world administrative boundaries [33], and world exclusive economic zones [34].

Ecuador is located on the northwestern coast of South America, comprising a continental (mainland) area and an insular region (the Galapagos Islands, located 1,000 km to the west) (Fig 1). The mainland borders Colombia to the north, Peru to the south and east, and the Pacific Ocean to the west, while the insular region borders only Costa Rica to the north [35]. Ecuador exercises sovereignty over an exclusive economic zone extending 200 nautical miles (370 km) from both coastlines [36,37], covering ~1,092,000 km²—nearly four times the national land area (257,217 km²) [38]. Ecuador’s artisanal fishing fleet is among the largest in the Eastern Tropical Pacific [8]. According to the SRP [39], nearly 46,000 fiberglass and wooden fishing boats operated in Ecuador a decade ago, providing employment for approximately 60,000 artisanal fishers targeting a variety of marine resources. Fishers specialize by region and target: some harvest crustaceans and bivalves; coastal fleets pursue small pelagics, demersal fish, and cephalopods; oceanic fleets target large pelagics (tunas, sharks) using longlines [40,41]. This stratification implies that fishing practices vary among villages and port areas depending on their proximity to fishing grounds, habitat types, and the distribution of targeted species [11]. For example, Esmeraldas, Manta, Puerto Lopez, Santa Rosa from Salinas, and Anconcito serve as primary ports for landing large pelagic species caught with longlines, whereas the remaining 261 fishing towns and villages are associated with the capture of smaller coastal resources using gillnets, deep-sea hook and line methods, and hand collection [11]. It is estimated that 18,599 fishers operate from Manabi and 7,688 from Santa Elena [39]. According to Martínez-Ortiz, Aires-da-Silva [8], nearly half of Ecuador’s artisanal fishers focus on resources other than large pelagic species, which suggests that approximately 9,300 and 3,844 fishers in Manabi and Santa Elena, respectively, could be employing gillnets, spearfishing, and deep-sea longlines.

### Data collection

From April to September 2023, we visited 22 small fishing villages in the provinces of Manabi and Santa Elena, located along the mainland coast of Ecuador (Fig 1). At each location, we contacted the leaders of the local fishing associations to request permission to interview their colleagues who primarily use gillnets, handlines, bottom-set longlines, and other fishing gears, excluding surface longlines. To ensure that we reached all available community members involved in nearshore fisheries, we complemented the information provided by community leaders with a snowball sampling peer-referral approach [42]. Additionally, we gathered data on the total number of registered fishers by asking association/union/cooperative leaders about their membership and reviewing available information published by the national fishing authorities (Table 1).

**Table 1:** Estimated sampling effort required for different numbers of fishers per fishing village, as recorded by various sources.

Fishing village	Fisher’s national census (2013)^1^	Licensed fishers (2023)^2^	Fisher’s national census (2024)^3^	Fishers according to leaders^4^	Estimated sampling effort
***MANABI***					
Cojimies	451	724	388	700	84 (80/88)
Cañaveral	20	25	39	50	24 (17/33)
La Chorrera	230	231	128	750	71 (56/88)
Jama (Bellavista and Don Juan)	272	358	633	---	79 (73/86)
Puerto Cabuyal	22	24	60	65	29 (18/39)
Canoa	149	181	210	300	67 (60/75)
Bahia de Caraquez	374	371	---	400	79 (79/80)
San Clemente	260	272	444	400	77 (72/82)
Crucita	352	471	62	---	66 (38/82)
Jaramijo	3,102	1,224	1,448	---	94 (92/97)
Santa Rosa (M)	110	99	22	---	40 (18/52)
Puerto Cayo	323	393	341	---	78 (76/80)
Machalilla	741	229	179	200	72 (64/88)
Puerto Lopez	1,109	531	521	---	87 (84/92)
***SANTA ELENA***					
San Pedro	45	215	133	1,000	62 (31/91)
Ayangue	196	91	32	250	52 (24/71)
Jambeli	184	108	255	400	67 (52/80)
Salinas (and Chipipe)	110	39	---	70	41 (28/52)
Santa Rosa (SE)	1,984	507	338	1,500	87 (77/95)
Anconcito	1,603	554	641	---	88 (85/94)

The total number of fishers per location was obtained from: 1, SRP (36); 2, SRP (45); 3, FENACOPEC (46); and 4, Fishing association leaders of Manabi and Santa Elena (47). None of the sources differentiates by gear type or fishing method. The estimated sampling effort is presented as the rounded mean, with minimum and maximum values shown in parentheses, calculated from the different sources reporting the total number of fishers per village. Fishing villages are listed in latitudinal order.

To estimate the required sampling effort per village, we followed Yamane [43] and applied the formula:


$$n=\frac{N}{\left[\text{1}+N(e{)}^{\text{2}}\right]}$$

Where *n* is the sample size, *N* is the population size, and *e* is the level of precision (±10%). This parameter, also referred to as sampling error, represents the range within which the true population value is expected to fall [44]. Since no official up-to-date information was available for the 22 villages, population size values for the number of fishers per village were obtained from various published sources and from the knowledge provided by fishing leaders in each community. These sources provided a population size ranging from 20 to 3,102 fishers [39], 24–1,224 [45], 22–1,448 [46], and 50–1,500 [47], which then produced an overall estimated sampling effort for fishers targeting coastal resources that ranged from 24 to 94 individuals per village, and from 380 (Manabi) to 360 (Santa Elena) fishers per province (Table 1). These values represent the unweighted average of the sampling efforts calculated from each source, shown along with their maximum and minimum values as a measure of their variability. No information was available regarding the number of fishers using surface longlines at each site, which limited our ability to calculate the required sampling effort based on the actual number of fishers using the other fishing gears relevant to the scope of this study.

Fishers were individually contacted and invited to participate in interviews designed to collect information on: i) demographic, experience, and occupational data; ii) use of fishing gear and targeted species; iii) their decadal perception of changes in their top three targeted species (if any); iv) decadal changes in the spatial extent of their fishing grounds; and v) perceived factors influencing those changes (used questionnaire form available in Supporting Material S1 Appendix). In the first section, fishers were asked to provide information on gender, age, years of fishing experience, fishing village, type of fishery (oceanic or coastal), fishing depth, and boat type. The second and third sections aimed to determine whether they had used specific fishing gears, identify their top three target species, and capture any general perceptions regarding catch trends. Since this study focused on depicting changes through time, no predefined set of species was provided before the interview, but all fishers were asked to share information about the main species they were targeting and were familiar with. The fourth and fifth sections explored the reasons behind those perceptions, if any. Lastly, fishers were asked to describe the extent of their fishing grounds and how they had changed over time. All questions addressing decadal changes referred to the 1980s, 1990s, 2000s, and 2010s. The current decade (2020s) was excluded to avoid potential bias introduced by the COVID-19 pandemic’s effects on fishing dynamics starting in 2020.

The design of the decadal variation in catches section followed the approach of Peñaherrera-Palma, van Putten [23] in which fishers were presented with five predefined categorical scores: major decline (MD), decline (D), stable (S), increase (I), and major increase (MI). Fishers were then asked to assign a percentage based on how much each categorical score meant to them (e.g., “decline” may equate to 25% decrease in abundance, while “major increase” might correspond to a 70% increase). The spatial evaluation section was based on the methodology of Beaudreau and Whitney [24], and involved asking fishers to draw their fishing grounds per decade on maps of coastal Ecuador and the Galapagos Islands, if their fishing activities extended offshore. Decade-specific maps were prepared using a reference map of Ecuador and the Galapagos, projected in the World Geodetic System (WGS 1984; EPSG code 4326).

### Data analysis

A semi-quantitative virtual abundance change model was applied to estimate the decadal variation in the virtual catches of target species, following the analytical framework published by Peñaherrera-Palma, van Putten [23]. This estimation, hereafter referred to as virtual catch change (VCC), begins in the earliest decade (1980s) with an initial standardized catch value of 1. Subsequent deficits or surpluses in catches were calculated for each decade by converting categorical trend scores and perceived percentage changes into a virtual abundance index. The model calculates catches for subsequent decades using the equation:


$$V_{ka}=\;X_{k-\text{1}}\;\pm \left(X_{k-\text{1}}*\;Y_{Zka}\right)$$

Where *V* is the virtual catch value estimated for decade *k* and fisher *a*. *X* is the virtual catch value of the decade *k-1*. *Y* is the percentage of perceived catch change of the corresponding categorical score *Z* for decade *k* and fisher *a*. When *k* is equal to 1980s, *X*_*k-1*_ is equal to the initial virtual catch, and when *k* is not equal to 1980s, *X*_*k-1*_ represents the virtual catch value of the previous decade (See Supporting Information S2 Appendix for a guide to the detailed mathematical calculations of these values). This method addresses cognitive shifting baselines due to varying fisher experience by weighting initial catches starting after the 1980s using the average virtual catch from the previous decade. A logic rule adjusts the abundance based on the trend score: stable maintains the prior value, declines subtract a percentage, and increases add a percentage [Full mathematical explanation available in [23]. The resulting VCC values were then used to construct temporal trend plots per gear type and species, and to associate each decadal value with its corresponding fishing ground (as explained below). The differences in VCC values between decades were calculated by measuring how much the value in Decade x (new value) changed relative to a previous Decade y (reference value) using the following equation:


$$Relative\;change=\;\frac{New\;value-Reference\;value}{Reference\;value}$$

Spatial data on fishing grounds were scanned, digitized, and converted into vector polygons using QGIS software [48]. Each polygon represented a fisher’s reported fishing area for a specific decade and was assigned a unique code to link it with the corresponding interview and VCC data. The spatial analysis was conducted in three stages. First, each polygon was assigned a value of 1 and aggregated to create a fishing effort raster layer, used to delineate the overall spatial extent and identify core fishing areas across all decades and per decade. The resulting raster layers were normalized on a scale from 0 to 1. Second, the distance from the centroid of each polygon to the corresponding port of origin was calculated using the ‘dist2Line’ function in the “geosphere” package [49] in R software [50]. Third, the initial value of 1 for each polygon was replaced with its corresponding VCC value (per fisher and decade) to produce a raster layer per decade, representing spatial averages of VCC. These raster layers were normalized from −4 to 4. All raster analyses were conducted using a uniform grid resolution of 0.01° and processed using the “raster” package [51] in R software. All maps were plotted over a basemap of the political and administrative division of Ecuador prepared by the National Secretary of Limits of Ecuador [32] and freely accessible through the National Institute of Statistics and Census Geoportal [52], the world administrative boundaries (countries and territories) available at Opendatasoft [33] under an Open Government License 3.0, and the world exclusive economic zones published by the Flanders Marine Institute [34] under CC-BY license.

### Ethical statement

This evaluation was conducted at the request of the fishing cooperatives in mainland Ecuador, and the designed interview was adapted with their input in accordance with previously approved protocols. Before the start of each interview, all fishers were informed of the objectives of this study and asked for their verbal consent to participate. No personal information was recorded during the evaluation, and thus, the collected data was analyzed anonymously.

## Results

A total of 1,436 fishers were interviewed regarding the status of their fisheries resources, as well as perceived changes in fishing grounds and catches over the past four decades (Table 2). Of these, 275 declined to participate, resulting in 1,161 completed interviews for analysis. The average age of fishers was 43.1 years (range: 15–82), while their mean fishing experience was 26.4 (range: 5–70). The oldest fishers were interviewed in Salinas and Chipipe (82 y.o.), Jaramijo and La Chorrera (78 y.o.), while the youngest were from Cojimies, Jama, Puerto Cayo, Ayangue (17 y.o.), Puerto Lopez (16 y.o.), and San Clemente (15 y.o.). The number of fishers who reported fishing activity during each decade was as follows: 350 in the 1980s, 608 in the 1990s, 914 in the 2000s, and 1,125 in the 2010s.

**Table 2:** Number of contacted and interviewed fishers per location.

Fishing village	Contacted fishers	Interviewed fishers	Target sampling effort	Sample size coverage	Age	Age range	Experience	Experience range
***MANABI***								
Cojimies	79	60	84	71%	46.9	17-77	31.5	7-70
Cañaveral	49	41	24	164%	50.7	27-77	27.5	5-57
La Chorrera	71	60	71	85%	43.3	19-78	24.5	5-70
Jama (Bellavista y Don Juan)	120	107	79	135%	38	17-74	21.6	5-50
Puerto Cabuyal	27	27	29	93%	40.6	18-70	22.1	5-59
Canoa	64	57	67	85%	39.7	18-77	20.9	5-61
Bahia de Caraquez	81	65	79	82%	48.3	22-77	29	5-70
San Clemente	75	54	77	70%	44.5	15-73	27.7	5-57
Crucita	137	112	66	170%	41.5	18-69	24.3	5-57
Jaramijo	45	26	94	28%	44.2	22-78	27.2	5-60
Santa Rosa (M)	52	24	40	60%	35	18-60	19	5-37
Puerto Cayo	67	49	78	63%	38.9	17-73	22.3	5-50
Machalilla	73	56	72	78%	43.4	20-74	27.4	5-57
Puerto Lopez	135	107	87	123%	46	16-77	28.1	5-63
***SANTA ELENA***								
San Pedro	58	51	62	82%	46.5	18-73	32.4	5-60
Ayangue	50	48	52	92%	46.8	17-76	32.3	5-61
Jambeli	47	30	67	45%	47.5	23-77	29.8	5-55
Salinas y Chipipe	42	42	41	102%	48.9	16-82	33.7	5-67
Santa Rosa (SE)	78	69	87	79%	46.1	19-70	26	5-63
Anconcito	86	76	88	86%	41.2	17-72	24.6	5-60

**Fig 2:**
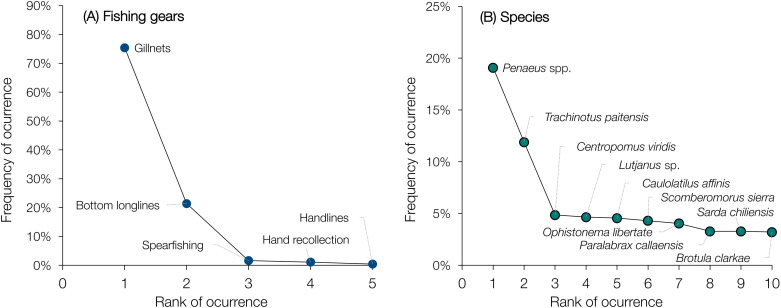
Ranking of fishing gears and targeted species based on their frequency of occurrence among respondents. **(A)** Reported fishing gears and their relative importance in coastal artisanal fisheries. **(B)** Top ten most frequently targeted species across all decades. The remaining 82 species are not shown due to their limited contribution (<0.03%) to the overall species assemblage.

Nearly all interviewed fishers (99.4%) operated small-sized boats ranging from 3 to 9 meters in length. Most (87.7%) reported having experience fishing in both inshore and oceanic areas, at depths ranging from 0 to 900 meters. A smaller portion (12.7%) operated exclusively offshore, with an average fishing depth of 92 meters, reaching down to 320 meters. Gillnets were the most used fishing gear among fishers (75.4%), followed by bottom longlines (21.4%), spearfishing (1.6%), hand gathering (1.2%), and handlines (including rod and reel; 0.4%) (Fig 2A). Over the past four decades, fishers reported targeting a total of 92 species. The most frequently mentioned included shrimps (*Penaeus* spp.), paloma pompano (*Trachinotus paitensis*), white snook (*Centropomus viridis*), snappers (*Lutjanus* spp.), bighead tilefish (*Caulolatilus affinis*), Pacific sierra (*Scomberomorus sierra*), Pacific thread herring (*Opisthonema libertate*), southern rock bass (*Paralabrax callaensis*), Eastern Pacific bonito (*Sarda chiliensis*), and Pacific bearded brotula (*Brotula clarkae*) (Fig 2B). Fishers also reported a steady increase in the number of targeted species per decade: 76 species in the 1980s, 85 in the 1990s, 89 in the 2000s, and 91 in the 2010s.

**Fig 3:**
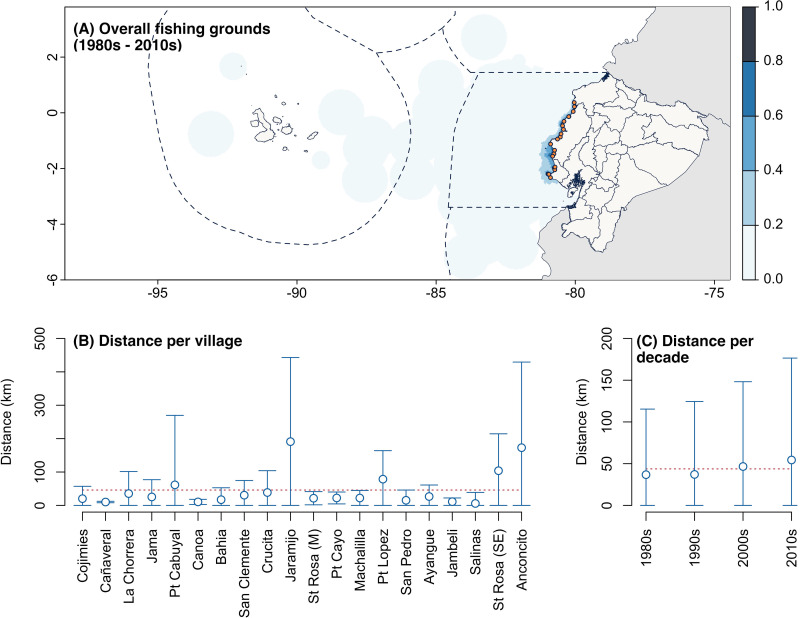
Location and distance of fishing grounds from the port of origin, by fishing village and decade. **(A)** Spatial concentration of fishing grounds reported by nearshore artisanal fishers across all decades. The color gradient shown in the map and the scale indicate the percentage of spatial overlap among fishing grounds, normalized from 0 to 1, calculated from the sum of all fishing grounds across decades. **(B)** Variation in the distance of fishing grounds from the coast per fishing village, and (C) per decade. Distances were calculated by measuring the distance between the centroid of each fishing ground polygon and its corresponding port of origin. The red dotted line in panels B and C represents the mean distance across all villages and decades. Basemaps: Political and administrative division of Ecuador [32], world administrative boundaries [33], and world exclusive economic zones [34].

Fishers reported operating in fishing grounds ranging from the mainland coast of Ecuador to offshore waters around the Galapagos Islands (Fig 3A). Core fishing grounds, defined as areas where 60% to 100% of interviewed fishers operated, were concentrated within 15 km (8 nm) of the mainland coast. The average distance of all fishing grounds from their port of origin was 46.5 km, though this distance varied considerably across fishing villages (Fig 3B). For example, fishers from Salinas, Cañaveral, Canoa, Jambeli, and San Pedro operated relatively close to shore, with average distances ranging from 5.8 to 15.3 km (median: 1.5 to 10.1 km). In contrast, fishers from Puerto Lopez, Santa Rosa (SE), Anconcito, and Jaramijo reported operating farther offshore, with average distances between 78 and 191 km (median: 38 to 75.5 km) (Fig 3B).

**Fig 4:**
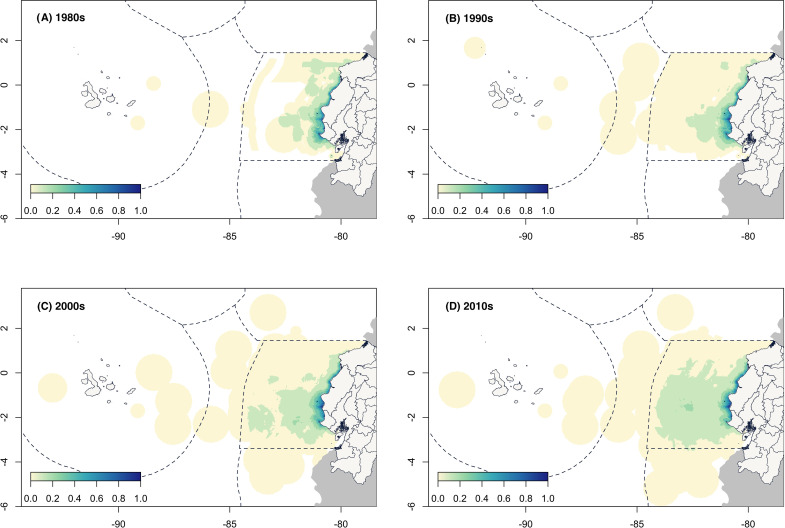
Decadal changes in the spatial distribution of fishing grounds. Maps show the normalized distribution of fishing grounds reported by fishers per decade. The color gradient represents the proportion of spatial overlap among fishing grounds, scaled from 0 to 1. Basemaps: Political and administrative division of Ecuador [32], world administrative boundaries [33], and world exclusive economic zones [34].

The average distance of all fishing grounds has steadily increased over time (Fig 3C). In the 1980s, fishing grounds were located at a mean distance of 36.8 km (median 12.2 km), in the 1990s at a mean distance of 37.1 km (median 12.9), in the 2000s at a mean distance of 46.6 km (median 14.5) and in the 2010s, at a mean distance of 54.4 km (median 15.7). This expansion was also observed from the spatial variation in fishing grounds per decade as drawn by fishers (Fig 4). While the core fishing grounds remained nearshore throughout the decades, the extent of all fishing grounds consistently increased until they covered the entire Ecuadorian mainland exclusive economic zone and extended to the insular exclusive economic zone around the Galapagos Islands. Specifically, this was mainly driven by consistent increases reported in Anconcito, Crucita, Jama, Jaramijo, La Chorrera, Machalilla, Puerto Cayo, Puerto Lopez, Salinas, Santa Rosa (M), and Santa Rosa (SE). Fishers from other villages reported relatively stable or marginal reductions in distance to port over the last decade.

**Fig 5:**
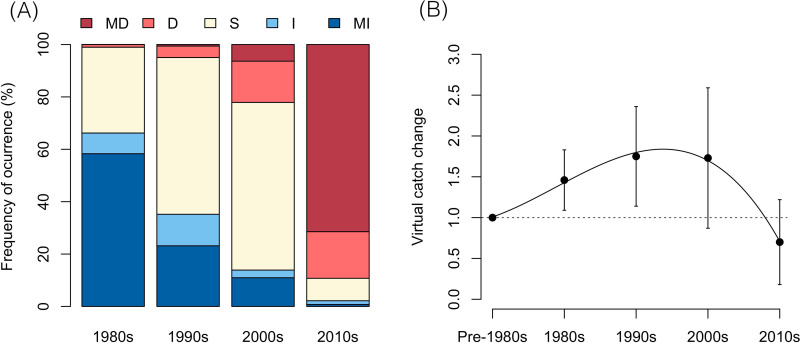
Summary of (A) the obtained trend scores and (B) the VCC analysis regarding the perceived changes in catches per decade. Positive categories include the obtained frequency of increase (I) and major increase (MI) scores, and negative categories, decrease (D) and major decrease (MD) scores. The stable score is represented by the letter **S.** The VCC analysis shows the mean (black dot), the standard deviation (error bars), and an adjusted 3-order polynomial regression (black line).

Most fishers (96%) reported that they have perceived significant changes in the number of fish they have caught in the last four decades, out of which 99.8% agreed that these changes have been negative. A closer look at their perceptions per decade shows that the polarity of this has not been the same through time (Fig 5A). Most fishers (~66%) acknowledged positive trend categories (increase and major increase) during the 1980s, with an inversion of their perception during the 2010s, in which a significant perception of negative trends (~89%) was conveyed.

Categories derived into VCC values produced estimations of catch changes in a very pronounced increase-decrease parabola relative to the pre-1980s (initial) baseline of 1. Catches exhibited a substantial increase during the 1980s, reaching 1.46, which further escalated to a peak of 1.75 in the 1990s, representing a relative increase of approximately 19.9% from the preceding decade of the 1980s (Fig 5B). This upward trend moderated slightly in the 2000s, with catches at 1.73, reflecting a modest decline of about 1.14% compared to the 1990s. However, a pronounced reversal occurred in the 2010s, when catches declined to 0.7, constituting a 30% decrease from the pre-1980s baseline and a more substantial reduction of approximately 59.5% from the 2000s.

**Fig 6:**
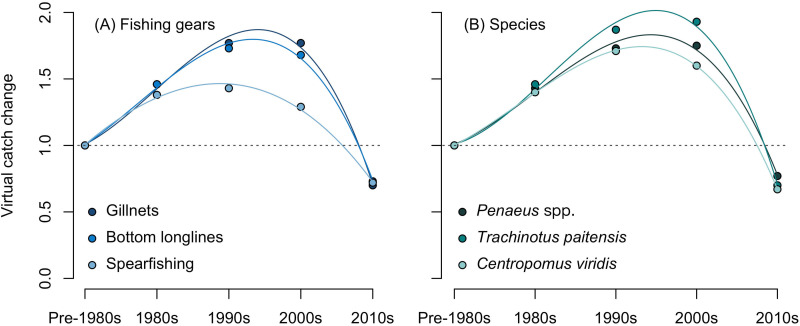
Virtual catch trend analysis for the top three fishing gears and target species. Plots show the mean (dots) and a 3-order polynomial regression (lines) for (A) gillnets, bottom longlines, and spearfishing; and (B) shrimps (*Penaeus* spp.), paloma pompano (*T. paitensis*), and white snook (*C. viridis*).

VCC estimations for the main fishing gears and targeted species showed a similar scenario (Fig 6). Catches with gillnets and bottom longlines increased by around 46% in the 1980s relative to pre-1980s levels (Fig 6A). This upward trend continued into the 1990s, with increases of about 21.2% and 18.5% compared to the 1980s. The 2000s showed stability, with no change for gillnets and a 3% decline for bottom longlines relative to the 1990s. In the 2010s, catches sharply declined by 42.9% and 37% relative to pre-1980s levels, and by 60.45% and 56.6% from the 2000s levels. The trend curve for spearfishing was less pronounced than gillnets and bottom longlines, yet it also followed the same type of increase-decrease parabola. Catches for this fishing gear were estimated to have decreased in the 2010s by 28% compared to the 1980s and by 44.2% compared to the 2000s. The top three targeted species followed the same parabola, with increases in the 1980s of about 40–46% relative to pre-1980s levels, and in the 1990s of about 21 to 28.1% relative to the 1980s (Fig 6B). In the 2010s, catches declined by 23–33% relative to pre-1980s levels, but from 56% to 63.7% relative to the 2000s.

**Fig 7:**
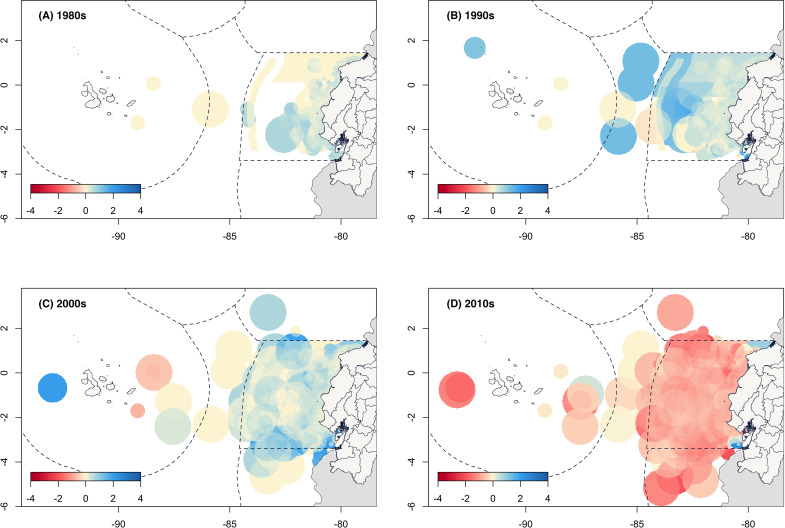
Spatial representation of the VCC across reported fishing grounds by decade. The color scale represents the additive value of fisher’s VCC responses. Basemaps: Political and administrative division of Ecuador [32], world administrative boundaries [33], and world exclusive economic zones [34].

The spatial analysis of the VCC also shows how fisher’s perceptions of catches have changed over time in relation to the expansion of their fishing grounds (Fig 7). For example, during the 1980s, most fishing grounds reported positive changes (from 0 to 200%) across the entire area. This scenario improved by the 1990s with the addition of areas further from the coast, resulting in positive catches at some fishing grounds that were up to two orders of magnitude higher compared to pre-1980s levels. By the 2000s, the spatial perception shifted toward more stable catch levels, while in the 2010s, fishers described stable to negative catches in most fishing grounds within the mainland exclusive economic zone. Only areas in the far north (Esmeraldas, bordering Colombia) and the far south (Santa Clara Marine Reserve, bordering Peru) showed positive VCC values. However, fishing villages in both regions were not evaluated; therefore, the spatial VCC metrics do not encompass the entire potential LEK of the fishers operating within those areas.

**Fig 8:**
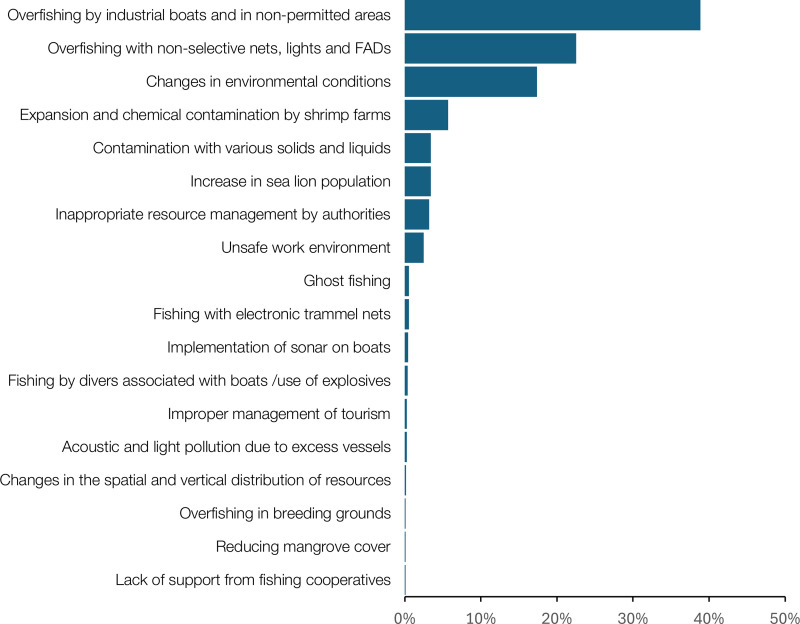
Ranking of perceived drivers of catch decline according to fishers.

During the interviews, fishers identified three main reasons for such changes as 1) overfishing by industrial vessels operating in unauthorized areas, particularly within the eight nautical miles artisanal fishing zone; 2) the use of non-selective fishing gears, such as “changas”, artificial lights, and fish aggregating devices (FADs); and 3) changes in oceanographic and atmospheric conditions (e.g., warmer waters and increased precipitation). In locations where fishing is associated with estuarine systems, such as Cojimies, Chorrera, and Bahia de Caraquez, fishers attributed declines in catches to mangrove deforestation for the construction of shrimp farms, and the discharge of chemicals from fish farming (e.g., metabisulfites). In southern fishing villages (from Puerto Lopez to Anconcito), fishers mentioned that the increase in the sea lion populations is affecting their normal fishing operation, as gillnets and lines are generally destroyed by the otariid species (*Otaria flavescens*) (Fig 8).

## Discussion

Artisanal fisheries have been largely underassessed, despite being one of the most important ocean activities that employ people and provide food for coastal communities [4]. In front of the continuous decline of inshore coastal marine resources and the lack of long-term fisheries monitoring data, providing information on coastal artisanal fisheries dynamics derived from fishers’ LEK is critical to ensure the sustainable management of coastal marine resources [21]. We used a semi-quantitative VCC framework to reconstruct the temporal and spatial dynamics of catches and fishing effort of artisanal fisheries in coastal Ecuador. Almost all fishers interviewed (~99%) indicated that catches have declined significantly. The perceived catch changes for all fisheries and species resembled a classical increase-decrease parabola, characteristic of depleted or near-collapsing resources. Simultaneously, fishers have not only diversified the species they target but have also expanded the spatial extent of their fishing grounds. This suggests that fishers now target a wider range of species and must travel farther to fish than they did in the 1980s, a result consistent with patterns observed in other fisheries globally [e.g., 53–55]. Our results highlight the importance of integrating fishers’ LEK as a key tool for monitoring catch trends and assisting the management of marine resources in data poor scenarios.

### Method validation and uncertainty

The value of incorporating fishers’ LEK in fisheries assessment and management is well documented globally [56,57]. However, uncertainty and biases can arise if the experiments are not adequately designed to address issues pertaining to the sample size [57,58], memory limitations [59], potential cognitive shifting baselines from varying levels of experience [60,61], or even the political and economic factors [62]. To overcome these issues, we took several considerations in the design and analytical framework adopted. First, the sample size was determined using the formula published by Yamane [43] with 10% precision to account for experience heterogeneity [57]. Discrepancies in the reported numbers of fishers per village highlight the lack of systematic fishing monitoring programs at these sites and for these fisheries [39,45–47], which resulted in a sample size coverage of 28% to 170% of the mean number of fishers per village. Except for Cabuyal (27 of 29 possible respondents interviewed), all villages exceeded the minimum sample sizes shown to produce reliable LEK results. For instance, Gray, Aminpour [63] demonstrated that a small sample of 33 fishers was sufficient to produce demographic trends closely matching those of the entire registered fisher population (170,000) and with estimates from formal scientific data. Additionally, Selgrath and Gergel [64] suggested that a sample size of nearly 1.1% of the total fishers’ population is adequate to capture almost 90% of the spatial distribution of fishing grounds. Following Martínez-Ortiz, Aires-da-Silva [8] and SRP [39], an estimated 26,288 fishers operate in Manabi and Santa Elena, with half (13,144) using gears other than surface longlines. Our 1,161 interviews thus represent 8.8% of the relevant population, well above established thresholds for robust spatial and temporal trend detection in comparable LEK studies. Although the overall sample is sufficient for regional-scale conclusions, the variable village-level coverage (28–170%) implies higher uncertainty for inferences specific to individual under-sampled communities.

Second, we adopted the virtual abundance change method proposed by Peñaherrera-Palma, van Putten [23]. This semi-quantitative approach systematically classifies, organizes, and assesses fishers’ memories of resource abundance based on their experience. Under this method, we asked fishers to select from predefined catch trend categories for their top three target species, instead of recalling the exact number or weight of fish caught per decade. By focusing only on their most targeted species, we ensured they provided the most accurate LEK on species they are most familiar with and economically interested in. This approach minimized issues related to numerical recall [65], misidentification, and inaccurate trend estimations from species away from their fishing experience [23,66,67], or being subject to polarizing topics that would make them evade answering truthfully to the interview [68]. The method also converts categorical scores into percentage changes and uses a cascade calculation to assess trends, reducing biases from complex mental calculations, memory lapses, and shifts in baseline perceptions over time. Furthermore, we ensure only data from fishers with more than five years of experience was included in the analysis. This is a critical step in fisheries evaluations since inexperienced fishers’ LEK holders are less likely to detect trends and signs of overexploitation of their resources [69]. Following this approach also ensures that our results are reproducible and comparable with other empirical studies, as demonstrated with similar semi-quantitative LEK methods [23,69–71].

### Temporal and spatial trends

Global increases in fishing effort, driven by technological advances, economic incentives, political priorities, and demographic pressures, have markedly intensified overfishing [72,73]. The proportion of sustainably fished stocks declined from 90% in 1974 to 64.6% in 2019, with 35.4% now overfished [74]. Studies have reported significant declines in catch per unit effort (CPUE) in Mozambique (by 91%), the Marshall Islands (by 88%), and West Africa (by 70%) [75–77]. Similarly, Peruvian small-scale fisheries showed slower catch growth relative to effort, reflecting depleted stocks [78]. In coastal Ecuador, our results suggest that fishers’ perceived parallel trends of intensified spatial and temporal fishing efforts amid declining primary resources. As reported by Alava, Lindop [79], Ecuadorian artisanal fisheries follow global trends driven by macro-level factors with direct impacts on fishers’ daily operations. Export demand for whitefish since the 1980s, coupled with insufficient industrial supply, pushed fishers to target lower-value and more distant species. Fuel subsidies and vessel upgrades in the 1990s enabled fishers to extend their range beyond the continental platform to access undepleted stocks [80,81], while industrial incursions into nearshore waters reduced local stock availability, forcing further offshore expansion and species diversification [46,79]. Our results reveal parallel intensification of spatial and temporal effort concurrent with declining primary resources, with catches reaching an asymptote in the 1990s–2000s, mirroring the critical threshold observed globally where expanded fishing capacity can no longer compensate for resource depletion [82]. This represents a troubling scenario for the Ecuadorian artisanal fisher, since small-scale fisheries are particularly more sensitive to collapse and socio-political pressure than larger industrial fisheries [83].

Since 2001, studies have reported that coastal resources in Ecuador were already declining or being overexploited [81]. Yet, these findings were mostly related to large-scale fisheries and pelagic species, which have been monitored for a greater length in Ecuador [79]. For example, the Public Institute for Aquaculture and Fisheries Research (*Instituto Público de Investigación de Acuicultura y Pesca, IPIAP*) has monitored and analyzed the landings for the purse-seiner fishing fleets since 1981 [84]. This multispecies fishery, the second most important in Ecuador after the tuna purse seine fishery, operates primarily with purse seine gears and is conducted by an industrial fleet of approximately 267 vessels, with an estimated annual catch of 278,000 metric tons [84]. The fishery mainly targets *Auxis* spp., *Cetengraulys mysticetus*, *Scomber japonicus*, *Decapterus macrosomma*, *Opisthonema* spp., and *Etrumeus teres*, which overlap strongly with artisanal catches. By 2018, IPIAP assessments classified all six stocks as overexploited, three of them critically so [85]. A similar scenario was reported by the industrial shrimp fisheries in Ecuador, whose catches have shown signs of decline since the 2000s [79], with the stock status of *Protrachypene precipua* shrimp assessed as overexploited, with evidence of high levels of fishing mortality [86]. However, these findings differ from subsequent assessments that show improvements in the population status of several species [84]. The discrepancies among assessments largely stem from inconsistent indicators and reference points, hindering direct comparisons and obscuring true population trajectories. While Ecuador has made notable progress in conducting stock assessments for several commercial species, standardized assessment protocols and routine reporting, including for bycatch species, remain essential for robust management [87].

### Management implications

The fishing dynamics documented through fishers’ LEK revealed a concerning situation for the coastal resources and the overall economic resilience of fishing villages in coastal Ecuador. Our results suggest that artisanal fisheries are facing reduced catches in nearshore fishing grounds, suggesting that the management of these areas (particularly within the eight nautical miles artisanal fishing zone) is not effectively improving the fishing conditions of coastal fishers. As reported for Peru, the continuous decline and collapse of marine resources can lead to widespread poverty among small-scale fishers [78], forcing difficult livelihood trade-offs that become unsustainable once stocks are depleted [88]. The collapse of fishing resources can induce fishers to seek different sources of income within the fisheries (from harvesting to processing) and outside of them (including construction, agriculture, and fuel sales) [11]. In extreme cases, it may lead to illegal activities, including the use of prohibited gear, poaching in marine protected areas, or involvement with criminal networks trafficking marine products or drugs [30]. The interference of criminal gangs during our interviews in certain areas of Santa Elena (Santa Rosa and Anconcito) and Manabi (Jaramijo and Puerto Lopez) highlighted the challenges fishermen currently face. These conditions increase the likelihood of illegal behavior when management remains ineffective and simultaneously complicate data collection, emphasizing the need for rigorous ethical safeguards and cautious interpretation of LEK in such vulnerable settings.

Interviewed fishers primarily attributed declining catches to industrial incursions into the artisanal-exclusive zone, widespread use of illegal gears, and environmental change. Changes in sea surface temperature in coastal Ecuador suggest that many less mobile coastal resources will soon be subjected to increased thermal stress due to the significant cooling of the Humboldt Current and the significant warming of the Panama Current [89]; while mobile species are expected to redistribute poleward or deeper in response to warming, deoxygenation, and acidification [90]. However, no specific analysis has been conducted on how the target species for Ecuador’s small-scale fisheries, examined in our study, respond to these changes. Given projections that climate change and overfishing will further reduce the proportion of stocks within biologically safe limits [90,91], policymakers must implement a multifaceted strategy that integrates environmental, economic, and social dimensions. Recommended measures include strict enforcement against destructive practices and industrial vessels within the eight-nautical-mile artisanal zone, ideally by redesignating it as a managed marine reserve with explicit conservation and sustainable-use objectives. Benefits to the fishing sector from implementing marine protected areas can enhance nutrient supply and catch potential by up to 20% in well-managed sites, while supporting ecosystem services such as habitat protection and resilience to overfishing [92,93]. Promoting sustainable fishing certifications can encourage responsible practices and improve market access for small-scale fishers in developing countries [94]. Furthermore, enhancing transparency in fleet ownership and operations, through tools such as VMS and AIS, is critical to address discrepancies and strengthen legal frameworks to combat illegal, unreported, and unregulated fishing [95], directly curbing industrial incursions noted by fishers. Additionally, international cooperation with neighboring countries, such as Peru, Colombia, and Costa Rica, is essential for effective regional fisheries management, given that cross-border fishing activities affect shared stocks of both small and large pelagic species [80].

Finally, the integration of fishers’ LEK with scientific data through participatory and transparent processes is fundamental to achieving fisheries sustainability [96]. This approach supports effective monitoring, adaptive management, and conflict resolution, while promoting sustainable and equitable fisheries [21]. For example, the incorporation of fishers’ LEK in a tropical inland fishery in Brazil contributed to the development of a successful management system that actively engaged both fishers and experts in resource monitoring, fostered horizontal knowledge exchange, and facilitated collaborative solutions to specific challenges [97]. In our study, fisher’s LEK facilitated the assessment of temporal trends in catches of the primary targeted species across the coastal regions of Ecuador, as well as the mapping of the previously unknown spatial dynamics of a fishery under no monitoring program. Small-scale fishers represent an important social sector of the Ecuadorian coast, whose contributions to community well-being have been historically underassessed and underrated. Ensuring the sustainability of their activities is essential not only for preserving their livelihoods, but also for safeguarding the health of coastal ecosystems in Ecuador.

## Conclusions

This study provides critical insights into the challenges facing Ecuador’s coastal small-scale fisheries and the communities that depend on them. By leveraging LEK, we documented a significant decline in fishery resources (30% lower than pre-1980s levels and 59.5% lower relative to the 2000s) and an expansion of fishing grounds since the 1980s, insights unattainable through traditional monitoring due to limited spatial and temporal data coverage. Fishers’ perceptions attribute these declines to industrial overfishing, non-selective practices, and environmental changes, emphasizing the need for sustainable management that integrates ecological and socio-economic factors. Our proposed pathway (e.g., enhanced transparency, regional cooperation, multi-stakeholder engagement, and targeted conservation), including the re-envisioning of the eight-nautical-mile zone as a managed reserve, offers an inclusive framework to address these issues. The collaboration between fishers and scientists demonstrates LEK’s ability to fill critical data gaps in data-poor fisheries, providing unique insights into local dynamics and serving as a vital decision-support tool. Continuous integration of LEK with scientific monitoring is essential for evaluating the efficacy of management interventions and facilitating adaptive management strategies, supported by robust institutional and policy-level frameworks to implement participatory monitoring strategies effectively.

## Supporting information

S1 Appendix Questionnaire form used to collect information from artisanal fishers. (DOCX)

S2 Appendix Virtual Catch Change modelling framework and dataset. The complete version of this dataset can be found at DOI: https://doi.org/10.5281/zenodo.17822727.
(XLSX)
