# Supplementary material for: Reconstructing the spatial and temporal dynamics of Ecuador’s artisanal small-scale fisheries from fishers’ perspective
Source: PLoS One. 2025 Dec 23;20(12):e0338495. doi: 10.1371/journal.pone.0338495 (PMC12725673; doi:10.1371/journal.pone.0338495)
Supplement: S1 Appendix — (DOCX) [file pone.0338495.s001.docx]

**Perception survey on the historical spatial changes in Ecuadorian artisanal fisheries**

*This survey aims to capture fishers’ perceptions of the historical status of fish stocks and fishing grounds used along the Ecuadorian coast. This study is conducted by MigraMar, a non-governmental organization, on behalf of the MPCEIP and MAATE. If you have any further questions, please do not hesitate to contact us. This study is confidential and will not collect or share any personal information from participants. .*

1. **Demographic information**

| Age: | Gender: | | Nationality: | | Type of fishery: Coastal/Oceanic | | Depth: |
| --- | --- | --- | --- | --- | --- | --- | --- |
| Years of fishing experience: | | Home port: | | Type of vessel: Wood/Fiberglass | | Tons/meters: | |

1. **Temporal trend**
2. What type of gear do you use for fishing? __________________________________________________________
3. Have you used the same fishing gear in recent years? Yes / No
4. If not, which fishing gear(s) have you used over the years?

1980. ___________________________________1990._____________________________________

2000. __________________________________ 2010.______________________________________

1. List the three most economically important species in your catches and the climatic season in which you catch them (cold or warm).

Spp 1. __________________________ Gear type: __________________. All year / Warm / Cold

Spp 2. __________________________ Gear type: __________________. All year / Warm / Cold

Spp 3. __________________________ Gear type: __________________. All year / Warm / Cold

1. Have you noticed any change in the amount of catch (number of fish) in recent years? Yes / No
2. How would you rate this change? Positive / Negative
3. Could you specify the observed changes for each species in the last four decades?

| Major decrease (MD), decrease (D), stable (S), increase (I), Major increase (MI) | | | |
| --- | --- | --- | --- |
| **Decade** | **Spp1:** | **Spp2:** | **Spp3:** |
| **1980’s** |  |  |  |
| **1990’s** |  |  |  |
| **2000’s** |  |  |  |
| **2010’s** |  |  |  |

1. What do "major decrease, decreased, increase and major increase" mean to you in percentage? Write the intensity of change from 0 to 100% according to each category.

Major decrease: ____________________________ Decrease: ____________________________

Major increase: _____________________________Major increase: _____________________________

1. Why do you think the abundance of your catch has changed over the past decades? List the most important (being the number 1 the most important).

(____) Change in water temperature. Currently warmer______; Colder________.

(____) Pollution of the sea. Type of pollution: ___________________________________

(____) Lack of support from fishing cooperatives

(____) Lack of management by the environmental authorities

(____) Overfishing using the same gear type.

(____) Overfishing using other gear types. Which ones? _______________________________________

(____) Other reasons not listed here. Which ones? ___________________________________________________

________________________________________________________________________________________

1. What alternatives do you propose to restore the productivity of artisanal fishing sector? ______________________________________________________________________________________________________________________________________________________________________________________________________________________________________________________________________________________________________________________________________________________________________________________________________________________________________________________________________________________________________________________________________________________________________________
2. **Spatial trend (if oceanic fishing). Mark with an X where applicable. Specify year and gear type.**


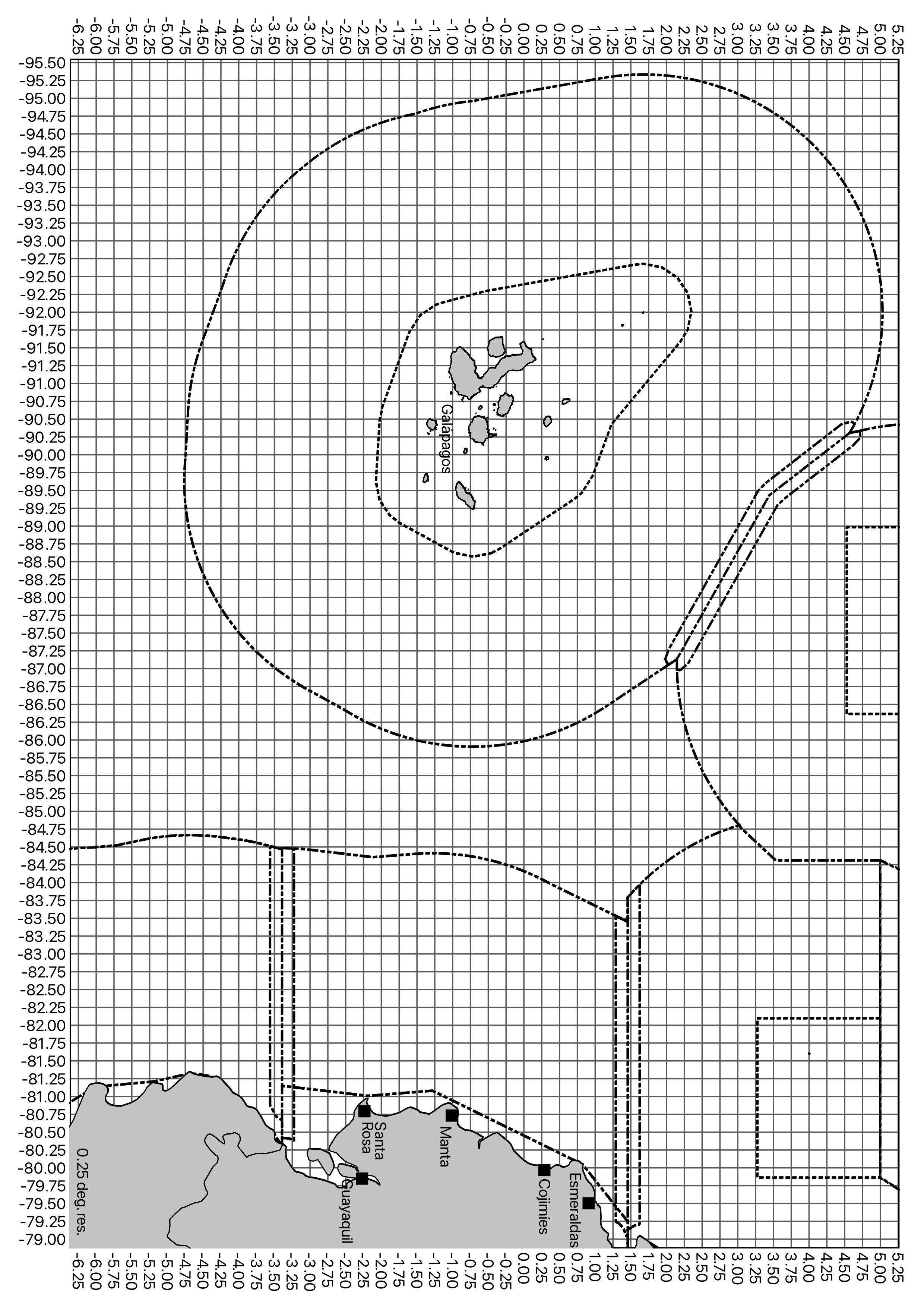


AÑO

1. **Spatial trend (if coastal fishing). Mark with an X where applicable. Specify year and gear type.**

**
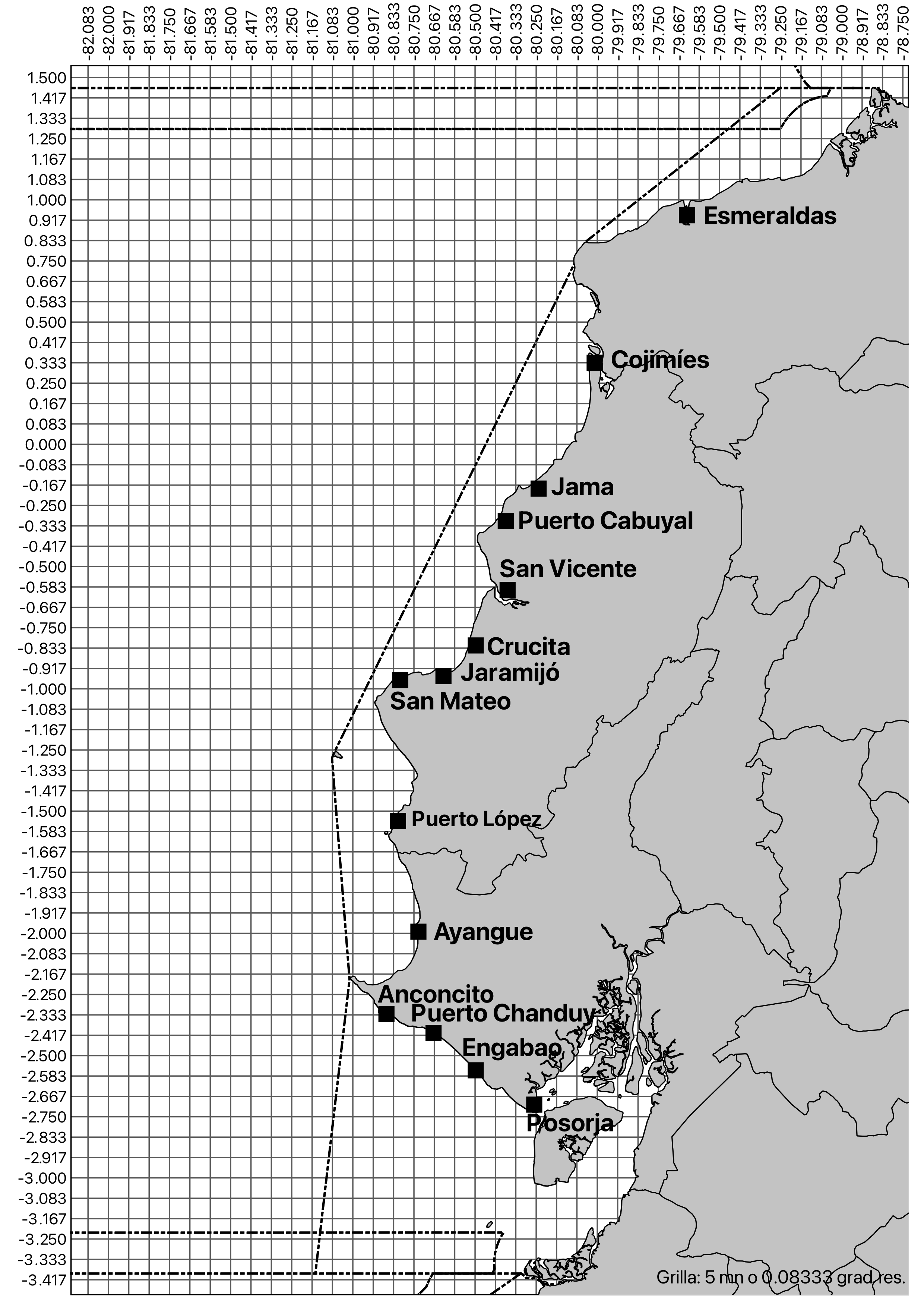
**

AÑO
